# Congenital tuberculosis in an extremely preterm infant conceived after in vitro fertilization: case report

**DOI:** 10.1186/s12884-017-1256-1

**Published:** 2017-02-20

**Authors:** Veronica Samedi, Stephen K. Field, Essa Al Awad, Gregory Ratcliffe, Kamran Yusuf

**Affiliations:** 1 Department of Pediatrics, Section of Neonatology, Cumming School of Medicine, University of Calgary, Calgary, AB Canada; 2 Department of Medicine, Section of Respiratory Medicine, Cumming School of Medicine, University of Calgary, Calgary, AB Canada; 3 Department of Radiology, Section of Neuroradiology, Cumming School of Medicine, University of Calgary, Calgary, AB Canada; 4 Rm 273, Heritage Medical Research Building 3330 Hospital Drive NW, Calgary, AB T2N 4N1 Canada

**Keywords:** Tuberculosis, Congenital, Placenta, Preterm neonate, Maternal, Case Report

## Abstract

**Background:**

Congenital tuberculosis is a rare manifestation of tuberculosis. The diagnosis is often delayed, especially in preterm neonates because of the non-specific clinical presentation and the lack of awareness of maternal disease prior to pregnancy.

**Case Presentation:**

We report a case of congenital tuberculosis in an infant born at 24 weeks of gestation to a mother who presented with uncontrolled seizures during preterm labor. Maternal diagnosis was initially made by placental pathology, and later confirmed by isolation of Mycobacterium tuberculosis in urine, gastric aspirates and sputum. Full screening was performed on the newborn infant, and both mother and infant were successfully treated for tuberculosis with a four drug regimen.

**Conclusion:**

Pregnancy can exacerbate latent tuberculosis and women originating from endemic areas are especially susceptible. The best way to prevent congenital tuberculosis is to have a high index of suspicion and identify and treat tuberculosis in pregnant women.

## Background

Despite advances in therapeutics and diagnostic techniques, tuberculosis (TB) continues to be a major infectious cause of morbidity and mortality worldwide. According to the World Health Organization, in 2015 there were 10.4 million new cases of TB worldwide of which 3.5 million were women and 1 million children [1]. TB continues to be amongst the top ten causes of death worldwide [1]. Although, over 95% of TB deaths occurs in low - and middle-income countries, the disease prevalence is increasing in developed countries due to migration from endemic areas [2]. While the burden of TB among pregnant women is equal to or greater than in the general population, many cases remain undiagnosed because of low threshold of suspicion and similarity of TB symptoms with physiological symptoms of pregnancy [3]. Even in high risk countries of Asia and Africa where severity of TB in pregnant women is confounded by HIV infection and malnutrition, screening for TB in preterm or term neonates born to mothers with confirmed or suspected TB infection remains inconsistent at best [4].

The burden of TB in children is not well established but is thought to account for 11% of all TB cases [2]. In 2014, 5% of the 1568 reported cases of TB in Canada were in children 14 years or younger with 2.9% in infants less than a year old [5]. Although data is limited, the prevalence of vertically transmitted TB from infected mothers to their off-springs can be as high as 16% [4]. Congenital TB can occur by hematogenous placental transmission of the organism from the mother to the fetus and also by ingestion of infected amniotic fluid or by direct contact with the organism during birth [6]. Although congenital TB is rare with about 350 cases reported in the literature, the numbers may be much higher as some cases could be missed as the diagnosis of congenital TB is challenging with some cases not diagnosed and others not reported [7, 8]. Initially based on autopsy findings, the criteria for diagnosis of congenital TB were revised in 1994 by Cantwell. These include the presence of tuberculous lesions in the infant with at least one of the following: lesions in the first week of life, a primary hepatic complex or caseating granulomas, tuberculous infection of the placenta or endometrium and exclusion of post-natal transmission of TB by screening contacts [8–10].

Genital TB, a major cause of infertility in women from endemic countries or belonging to high-risk ethnic groups, is also a risk factor for congenital TB especially I with increasing access to assisted reproductive technology including in vitro fertilization (IVF) [11–14]. We report a case of congenital TB in a 24-week gestation infant whose mother had genital TB and who was conceived by in vitro fertilization (IVF). To the best of our knowledge, only five cases of congenital TB following IVF have been described and of the 350 reported cases, very few have reported adequate pathological evaluation of the placenta.

## Case Report

A 37-year-old South Asian woman with no significant past medical history except for infertility and thalassemia minor underwent IVF for the first time. Infertility workup before IVF detected no abnormalities of the uterus or tubal lesions but endometrial biopsies were not performed. She had regular prenatal follow up, and used folic acid and prenatal vitamins. With the exception of intermittent vaginal bleeding noted since 10 weeks of pregnancy, her pregnancy was uneventful until 20 weeks of gestational age (GA) when she had spontaneous rupture of membranes. Fetal ultrasound (US) showed severe oligohydramnios. Repeat US at 23 weeks showed absence of the septum pellucidum and right talipes equino varus in the fetus. At 24 weeks GA, the mother presented with generalized seizures, went into precipitous labour delivering a male infant by spontaneous vaginal delivery with a birthweight of 590 g and APGAR scores of 4, 6, and 6 at 1, 5 and 10 min respectively.

Resuscitation of the infant in the case room included intubation, intermittent positive pressure ventilation with up to 100% oxygen and surfactant administration. High Frequency Oscillatory Ventilation (HFOV) was started in the case room due to the high oxygen requirements and absent chest movement.

**Fig. 1:**
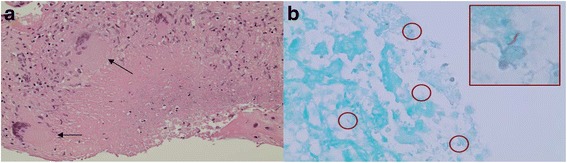
**a** Maternal surface of the placenta showing necrotizing granulomatous deciduitis. **b** Ziehl-Neelsen stain showing acid fast bacilli

**Fig. 2:**
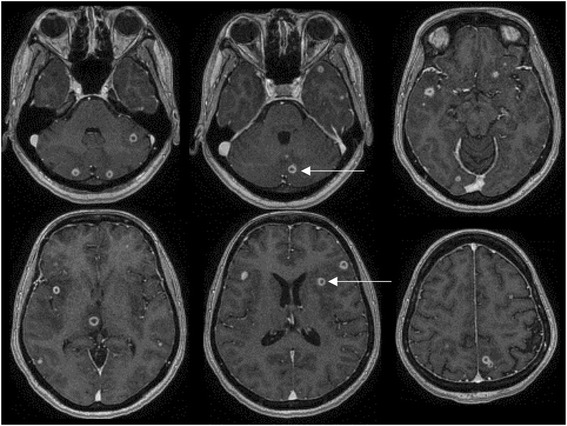
Maternal MRI showing small ring-enhancing lesions in both cerebral and cerebellar hemispheres

The mother was admitted to the Intensive Care Unit with continuous seizure activity for a diagnostic workup. Diagnosis of TB was made by placental pathology on day three post-delivery that showed necrotizing granulomatous deciduitis and sub-chorionitis with acid-fast bacilli (AFB) apparent on Ziehl-Neelsen staining (Fig. 1a, b). The findings were suggestive of placental involvement from chronic endometrial infection rather than recently acquired or haematogenous infection. The parents denied any family history of TB or contact with a known tuberculous patient. Anti-tuberculosus treatment was started while awaiting confirmation of the diagnosis. The diagnosis was confirmed by a positive Polymerase Chain Reaction (PCR) on placental tissue for Mycobacterium Tuberculosis and culture of the organism from sputum and urine after 15 and 17 days of incubation respectively. Her chest X ray was suggestive of military TB and CT scan of the chest confirmed the pulmonary involvement. An abdomen ultrasound was reported to be normal. Her brain magnetic resonance imaging (MRI) showed multiple small ring-enhancing cerebral and cerebellar lesions, consistent with an atypical infection such as TB (Fig. 2).

Immediately after the diagnosis of maternal TB and due to the complicated respiratory course of the infant (HFOV in case room, repeated doses of surfactant, presentation with Persistent Pulmonary Hypertension of Newborn (PPHN) within 24 h of life that required nitric oxide (iNO)), the infant was fully screened for TB.

Given the florid miliary and placental TB in the mother and the infant’s critical condition, he was started empirically on anti-tuberculous treatment on day 3 of life. Mycobacterial culture of blood, cerebro-spinal fluid (CSF), endotracheal aspirates, gastric aspirates, urine and stool were, however, negative. Since confirming the diagnosis of congenital TB is difficult and the associated high mortality and morbidity, it was decided to continue anti-tuberculous treatment. The infant was started on isoniazid (10 mg/kg/d), rifampin (20 mg/kg/d), pyrazinamide (30 mg/kg/d), and ethambutol (20 mg/kg/d) for 2 months with a plan for isoniazid and rifampin for a further 4 months. His course in NICU was complicated by severe respiratory insufficiency, radiological evidence of pulmonary interstitial emphysema and early signs of chronic lung disease (CLD).

The baby remained on HFOV for 25 days and required iNO support for 29 days. A course of dexamethasone was required to facilitate extubation to Continuous Positive Airway Pressure.

At 42 weeks of corrected GA the infant’s growth is appropriate, he has CLD requiring oxygen via nasal cannula and a brain MRI was reported to be normal. His eye examination and liver function tests are normal. The mother continues her anti-TB treatment and is doing well.

## Discussion and conclusions

Our case report highlights the need to raise awareness about the possibility of latent genital tuberculosis in an infertile woman and initiating timely anti-tuberculous therapy in newborns. Genital TB is a common cause of infertility in women from areas where TB is prevalent [12]. However, with increasing global migration, genital TB is being increasingly recognized in developed countries [2]. Although congenital TB due to maternal genitourinary TB is uncommon, the increasing availability of assisted reproductive technologies, especially IVF, has the potential to increase the prevalence of congenital TB [13, 15, 16]. Currently, IVF is one of the commonest treatment for infertility, and the number of babies conceived through IVF is increasing [14]. Presently, many women whose infertility was caused by genital TB choose IVF as an option to conceive [13, 16]. In developed countries, immigrants for endemic areas, such as the mother of our patient and HIV infected mothers in resource limited countries would be at high risk for TB. Screening for latent TB during pregnancy can, however, be problematic. The tuberculin skin test can be false positive in mothers who have received BCG vaccine and be false negative in mothers with HIV. Some investigators do not recommend chest X-ray in pregnancy and the AFB smear has a low sensitivity. AFB culture is time consuming and may not be easily available everywhere. The interferon-gamma release assays (IGRA) test is recommended by some investigators as the results are not affected by BCG vaccination or HIV status [17]. Thus, a high level of clinical suspicion has to be maintained in high risk populations with a thorough assessment including a detailed clinical history at the time of prenatal visits [3, 17]. Importantly, pregnant women with TB who are treated appropriately do not have increased levels of maternal or neonatal complications [17].

The infant in our case did not meet all of Cantwell’s criteria as we were unable to isolate mycobacterium tuberculosis from any of the biological samples. The diagnosis of congenital TB can, however, be challenging. Diagnostic tests for TB have extremely poor sensitivity in newborns and some investigators suggest that the diagnosis of congenital TB should be based on clinical criteria [9, 10]. The tuberculin test is universally negative in newborns and the IGRA is also usually negative as the T lymphocytes in newborns do not have the capacity to generate interferon-gamma in response to antigenic stimulation [7, 9, 11]. The clinical symptoms associated with congenital TB are non- specific and similar to those in viral or other bacterial infections [2, 6, 9]. Furthermore, radiological chest findings may be non-specific [2, 6, 11]. Mycobacterial smears and PCR detection can be negative in 30% of cases [7, 9]. However, a number of clinical features placed this infant at extremely high risk of having TB. The greatest risk of transmission to the fetus is miliary or bacillemic TB in the mother at the time of or just prior to delivery, like our case. Endometrial and placental granulomas were present and AFB were demonstrated in the placenta. The infant’s chest x-ray also deteriorated rapidly although it was difficult to ascertain whether this was due to prematurity or TB. The neutrophil count was low (1.2 × 10^9^/L) which has been suggested as a bad prognostic sign in congenital TB [11]. Importantly, mortality of untreated disease is 100% [9, 11]. Given these concerns, the infant was empirically treated for TB. Amongst the five cases of congenital TB following IVF reported by Flibotte, the organism could not be isolated from one infant [13]. Stuart et al. have reported a case after IVF where the organism was not detected in CSF, blood or gastric aspirate by PCR or culture but was present in a lymph node biopsy [15].

In summary, pregnancy can exacerbate latent TB infection. Women from high TB prevalence areas are especially at risk and those with genital TB may transmit the disease to the fetus. The diagnosis of congenital TB can be difficult as diagnostic tests are insensitive and the clinical signs and symptoms non-specific. The best way to prevent congenital TB is to maintain a high index of suspicion in women from high prevalence areas and to identify and treat TB in pregnancy. Multicentre trials to establish diagnostic tests including molecular tests such as PCR, with better sensitivity and specificity for TB are needed, especially in pregnancy and neonates.
